# Thrombotic thrombocytopenic purpura

**DOI:** 10.1007/s12254-018-0429-6

**Published:** 2018-08-17

**Authors:** Paul Knöbl

**Affiliations:** Department of Medicine 1, Division of Hematology and Hemostasis, Medical University of Vienna, Währinger Gürtel 18–20, 1090 Vienna, Austria

**Keywords:** Thrombotic-thrombocytopenic purpura, Thrombotic microangiopathy, ADAMTS13, Platelets, Von Willebrand factor

## Abstract

Thrombotic thrombocytopenic purpura (TTP) is a clearly defined entity of the thrombotic microangiopathies (TMA), a heterogeneous group of disorders characterized by microangiopathic hemolytic anemia with red cell fragmentation, thrombocytopenia and organ dysfunction due to disturbed microcirculation. TTP is characterized by a severe deficiency of ADAMTS13 (a disintegrin and metalloproteinase with a thrombospondin type 1 motif, member 13), an enzyme responsible for physiological cleavage of von Willebrand factor (VWF). Organ dysfunction can be severe and life-threatening, and immediate start of appropriate therapy is necessary to avoid permanent damage or death. Until recently, therapeutic options were limited to symptomatic measures, which were not standardized or based on high scientific evidence. In recent years, not only considerable progress has been made in better diagnosis of TTP, but also new therapeutic strategies have been established. Initial treatment is still based on plasma exchange and symptomatic measures to protect organ function, but new concepts (immunosuppression, targeted anti-VWF or anti-complement therapy, replacement with recombinant enzymes) have recently demonstrated impressive advantages.

## Introduction

Thrombotic thrombocytopenic purpura (TTP) is a well-defined entity of a heterogeneous group of disorders, the thrombotic microangiopathies (TMA). TMAs are characterized by microangiopathic hemolytic anemia with red cell fragmentation, thrombocytopenia and signs of organ dysfunction due to disturbed microcirculation. TTP is a rare disorder with an incidence of about 5 per million per year. It was first described in 1924 by Eli Moschcowitz, who reported a 16-year-old girl who died after acute onset hemolytic anemia, thrombocytopenia, petechiae, fever and severe neurological symptoms [1]. In 1982, Moake et al. recognized the abnormal composition of von Willebrand factor (VWF) multimers in the plasma of patients with TTP [2], and in 1998 Furlan et al. [3] and Tsai and Lian [4] identified ADAMTS13 (a disintegrin and metalloproteinase with a thrombospondin type 1 motif, member 13) deficiency as the pathogenic cause of TTP. In recent years, not only considerable progress has been made in the diagnosis of TTP, but also new therapeutic strategies have been established [5–7], including new drugs targeting specific parts of the pathophysiologic processes leading to TTP.

## Thrombotic thrombocytopenic purpura

The current pathophysiological concept understands TTP as a state of severe deficiency of ADAMTS13, which can be caused either by genetic abnormalities (congenital TTP) or by autoantibodies affecting function or clearance of ADAMTS13 (autoimmune TTP). Lack of ADAMTS13 leads to the persistence of UL-VWF MM (ultralarge VWF multimeres). In the presence of additional triggers causing shear stress and unfolding of VWF (pregnancy, infections, certain drugs, surgery, etc.) enhanced platelet aggregation with the UL-VWF MM occurs. These platelet aggregates affect the blood flow in the microcirculation and cause organ damage and clinical symptoms [8]. In TTP, the central nervous system is mainly affected [9], but other organs especially involved are the kidneys and the heart. VWF-rich platelet thrombi containing low/no fibrin can be found in the capillaries, both smaller and larger vessels, in histological samples of patients with acute TTP.

The determination of ADAMTS13 activity is essential in TTP, as only very low levels (below the detection limit of most assays) are associated with TTP. As soon as at least low ADAMTS13 activity is detectable (i. e. >10%), VWF can be cleaved and TTP will not occur. Current laboratory methods (FRETS-VWF73 or GST-VWF73) have a low detection limit, great accuracy and fast throughput, older methods (collagen-binding assay, VWF degradation assays) are essentially outdated. Measurement of ADAMTS13 antigen may reveal detectable levels, as ADAMTS13 protein or immune complexes are also detected. Other assays are used to detect antibodies binding to ADAMTS13 and to distinguish between congenital and acquired TTP. Functional inhibitors of ADAMTS13 are quantified by dilution series according to the Bethesda method used for FVIII inhibitors [10].

### Congenital ADAMTS 13 deficiency (Upshaw–Schulman syndrome)

Numerous mutations and polymorphisms in the ADAMTS13 gene are known [11, 12], leading to a severe reduction of ADAMTS13 activity (below the detection limit of most assays, i. e. <1 U/mL). Congenital (familial) TTP (OMIM No. 274150; http://www.ncbi.nlm.nih.gov/omim) can manifest early in childhood, but also later in life (in women often during the first pregnancy) and tends to relapse. Bouts of TTP can be triggered by factors associated with high shear rates (infections, pregnancy, drugs, etc.). Individuals with higher endogenous ADAMTS13 activity are usually safe and never experience TTP [13].

Diagnosis is performed by demonstrating the lack of ADAMTS13 activity with an assay of appropriate sensitivity, ruling out anti-ADAMTS13 antibodies (by testing for ADAMTS13 inhibitors and ADAMTS13-binding antibodies) and by sequencing the ADAMTS13 gene. ADAMTS13 antigen (but representing dysfunctional protein) may be detectable in the plasma, depending on the type of mutation. An international registry on patients with Upshaw–Schulman syndrome (www.clinicaltrials.gov; NCT01257269) is currently collecting all available cases of this rare disorder [14].

### Acquired ADAMTS13 deficiency (autoimmune TTP)

Autoantibodies targeting ADAMTS13 either inhibit the function or enhance clearance. Several factors are known as triggers of the autoimmune process, such as infections with viruses (Epstein–Barr virus, cytomegalovirus, HIV, etc.) or other pathogens, malignancy, certain drugs, other concomitant autoimmune diseases, pregnancy, but in many cases no underlying disorder is found. During the oligo-/polyclonal immune reaction several types of autoantibodies can be formed over time [15], and the detection of these antibodies is dependent on the assays used [16, 17].

Diagnosis is performed by demonstrating the absence of ADAMTS13 activity with an assay of appropriate sensitivity, and the detection of anti-ADAMTS13 antibodies (by testing for ADAMTS13 inhibitors and/or ADAMTS13-binding antibodies) confirms the immunologic nature, but sensitivity of the available assays is moderate and there may be false-positive and false-negative results. ADAMTS13 antigen (representing ADAMTS13 bound in immune complexes) may be detectable [18, 19].

## Clinical symptoms

The key clinical symptoms of TTP are, as for all types of TMA, symptoms of Coombs negative hemolysis with red cell fragmentation (recognized by anemia, elevated LDH, free serum hemoglobin, reticulocyte and schistocyte counts, reduced haptoglobin levels, and hemoglobinurea), consumption thrombocytopenia and signs disturbed microcirculation. The symptoms of organ dysfunction are often nonspecific and very variable [5, 7, 10]. Brain hypoperfusion can cause a broad variety of unspecific neurological symptoms, ranging from headache, blurry speech, dizziness, or agitation to stroke, amaurosis, epileptic seizures or coma. Renal involvement leads to increased serum creatinine, oligo- or anuria, and hemolysis-induced hemoglobinuria. Hemolysis may also cause jaundice and signs of anemia; thrombocytopenia is only sometimes associated with purpura, and bleeding is rare. Cardiac involvement with increases in cardiac enzymes (troponin, creatine kinase of cardiac isotype), patterns in the electrocardiogram resembling myocardial hypoperfusion/infarction, or arrhythmia, and the development of myocardial failure, is a dangerous complication of TTP, which may lead to immediate death of the patient [20]. Other organ manifestations may include lung (gas exchange problems, lung infiltrates), pancreas (increased enzymes, diabetes) or gut [21].

## Initial diagnosis

A patient presenting with a bout of TTP is one of the most challenging hematological emergencies [6–8]. Immediate appropriate diagnostic procedures (Table 1) are necessary to clearly identify TTP and to distinguish this from other forms of TMA. Careful examination of the patient’s history will reveal possible causes of TMA. Before any therapeutic approach is started, the patient should be screened for the eligibility for a clinical trial, and necessary procedures need to be performed before start of therapy (informed consent, sample acquisition, randomization, etc.). Moreover, samples of patient’s plasma, serum and blood cells should be obtained to be either stored for later analysis (biobanking) and immediate analysis of ADAMTS13-, VWF-, and complement-related parameters. Assessment of organ function should be performed with appropriate methods. In any case, until diagnosis has been confirmed, TTP should be suspected and immediate appropriate therapy with plasma exchange and steroids (Table 2) should be initiated without unnecessary delay to achieve the best outcome for affected patients [6–8].

**Table 1:** Diagnostic approach to acute thrombotic microangiopathy

Condition	Diagnostic tests
*Hemolysis*	Hemoglobin, red blood cells, indices
	Reticulocyte and schistocyte counts
	Lactate dehydrogenase, haptoglobin
	Direct antiglobin test (DAT; Coombs test)
*Thrombocytopenia*	Platelet counts; Immature platelet fraction
*Organ damage*	
Brain	Imaging: CT scan^a^, perfusion MRI^a^
	Electroencephalogram^a^
	S100 beta, neuron-specific enolase
	Neurocognitive testing^a^
Kidneys	Serum creatinine, glomerular filtration rate
	Urine output
Heart	Electrocardiogram
	Troponin, NT-proBNP
	Echocardiography
Lung	Oxygen saturation, gas exchange
	Imaging: chest x‑ray, high-resolution lung CT scan^a^
Coagulation	Plasmatic coagulation assays
	Antiphospholipid antibodies
Pancreas	Blood glucose, serum lipase
*Specific diagnosis*	
General	Biobanking, sampling for possible clinical trials
	Blood group typing
	Pregnancy test^a^
	Tests for viral infections (HIV, hepatitis B and C)
	Urine analysis
	Thyroid function tests
Thrombotic thrombocytopenic purpura (TTP)	ADAMTS13 activity, antigen
	Anti-ADAMTS13 antibodies and -inhibitor
	VWF:Ag, VWF:activity, VWF:RCo, VWF:CBA, VWF multimeric pattern
	ADAMTS13 gene analysis
Hemolytic uremic syndrome (HUS)	Tests for bacterial infection/toxins (E. coli, Shigella, etc)
	Complement C3 activation products, C4, CH50, APH50, C5a, terminal complement complex, CFH antibody
	Complement factors gene analyses
*Medical history*	Concomitant and previous diseases
	Underlying conditions (cancer, infections, systemic diseases, transplantation, pregnancy, surgery)
	Drugs, medication
	Family history

*CT* computerized tomography; *MRI* magnetic resonance imaging, *BNP* brain natriuretic peptide; *VWF* von Willebrand factor; *Ag* antigen; *RCo* ristocetin cofactor activity; *CBA* collagen binding activity; *CFH* anti-complement factor H; *HIV* human immunodeficiency virus; *ADAMTS13* a disintegrin and metalloproteinase with a thrombospondin type 1 motif, member 13; *CH50* total complement activity; *APH50* complement alternate pathway 50

^a^When clinically indicated and appropriate

**Table 2:** Therapeutic options for thrombotic thrombocytopenic purpura

Therapeutic option	Indication	Dose	Mechanism of action
*Established options*			
Plasma exchange	Initial therapy in all types of TMA Treatment of choice in autoimmune TTP	60–80 ml/kg/day	*Elimination* of autoantibodies, immune complexes, UL-VWF MM, sludge *Replacement* of ADAMTS13 and regularly composed VWF
Plasma infusion	Congenital ADAMTS13 deficiency (Upshaw–Schulman syndrome)	10–40 ml/kg every 2–3 weeks	*Replacement* of ADAMTS13
Corticosteroids	Autoantibody-induced TTP	1–2 mg/kg/day	Immunosuppression
Rituximab	Autoantibody-induced TTP	4 doses of 100–375 mg/m^2^/week	Immunosuppression
Immunomodulators (vincristine, MMF, cyclosporine, cyclophosphamide)	Autoantibody-induced TTP (3rd line immunotherapy)	As indicated	Immunosuppression
Anti-platelet agents (ASS, clopidogrel, prasugrel, ticagrelor)	TTP with severe organ damage	Clopidogrel: 75–150 mg/day	Inhibition of platelet aggregation
Splenectomy	Refractory TTP (after rituximab failure)	–	Unknown. Elimination of memory cells?
Supportive therapy	Anemia: RBC transfusion organ failure: ICU	–	(Details: see text)
*Future options*			
Caplacizumab^a^	Acute autoimmune TTP	10 mg/day sc	Blocking VWF A1 domains, competition with platelet GP Ib/IX
Recombinant ADAMTS13^b^	Congenital deficiency of ADAMTS13 (Upshaw-Schulman syndrome)	20–40 U/kg every 2–4 weeks	Replacement of ADAMTS13
Recombinant ADAMTS13^b^	Autoimmune TTP?	Unknown	Replacement of ADAMTS13 to overcome inhibitors
N-acetylcysteine^b^	Acute and chronic TTP	300 mg/kg/day	Cleavage of UL-VWF MM

*TMA* thrombotic microangiopathy; *TTP* thrombotic thrombocytopenic purpura; *UL-VWF MM* unusually large von Willebrand factor multimers; *RBC* red blood cells; *ICU* intensive care unit; *ADAMTS13* a disintegrin and metalloproteinase with a thrombospondin type 1 motif, member 13; *GP* glycoprotein

^a^Early access program available

^b^In development

## Therapeutic options for TTP

### Plasma exchange therapy

Plasma exchange (PEX) was introduced in the treatment of TTP by Rock et al. [22] and has improved survival from about 10 to 80–90%. During this procedure, the 1.5-fold plasma volume is removed and replaced by donor’s plasma (either fresh frozen single donor plasma or pooled, virus-inactivated plasma are acceptable replacement fluids; cryosupernatant is also used in some countries). During PEX, autoantibodies, UL-VWF MM, immune complexes and sludge are removed, and ADAMTS13 and VWF-MM of normal composition are replaced. In patients with TTP, daily PEX should be continued until platelet counts and LDH are normal and signs of hemolysis and organ dysfunction have resolved. In refractory cases and severe organ dysfunction, treatment intensity can be increased either by increasing the exchanged plasma volume or by performing PEX twice daily [6–8].

Patients with congenital ADAMTS13 deficiency usually respond rapidly and platelet counts normalize within a few days. Patients with secondary types of TMA (i. e. transplant-associated, some types of drug- or infection-induced TMA) will not respond to PEX, and treatment should not be continued except as a symptomatic measure for patients in poor condition.

### Plasma infusion

Therapy of congenital TTP is usually performed by plasma infusions to substitute for the missing enzyme. Even acute bouts with severe symptoms usually respond well to the infusion of plasma (20–40 ml per kg body weight). In patients with frequent relapses or smoldering disease regularly prophylactic plasma infusions (every 2–3 weeks) are necessary, as such patients often have low-grade, but detectable neurologic disturbance [23–25]. In autoimmune TTP, however, response to plasma infusions is poor [22] and should be used only to bridge the time to the start of sufficient PEX therapy.

### Immunosuppression

Immunosuppression with corticosteroids (i. e. prednisone, 1–2 mg/kg/day) is useful in cases of autoimmune TTP to suppress further antibody formation. In addition, even in other forms of TMA, steroids may be useful to reduce shear stress and improve endothelial function. Moreover, data from the Oklahoma Registry suggest that the number of PEX necessary to achieve remission and the incidence of complications of PEX were considerably reduced since the introduction of steroids in TTP therapy [26]. Steroids are usually maintained until hematological remission or until resolution of the autoimmune process, monitoring ADAMTS13 activity and anti-ADAMTS13 antibodies is helpful to guide therapy.

Several reports have been published on the effects of rituximab to eliminate anti-ADAMTS13 antibodies in patients with TTP [27–29]. Efficacy to eradicate the autoantibodies is high; 95% had a complete clinical and laboratory response within 1–3 weeks, and the effect usually lasts for more than 2 years. A randomized clinical trial (STAR trial; NCT00799773), however, has been terminated due to a low enrollment rate. Considering the high risks of permanent organ damage in relapsing or refractory TMA, the reported side effects of rituximab have to be weighed against the chance to obtain a long-lasting response.

Refractory or frequently relapsing patients (e.g., failure of steroids) are often treated with splenectomy or fourth line immunosuppressive treatment with vincristine, mycophenolate mofetil (MMF), cyclophosphamide, bortezomib, or bendamustine (all of them off-label and based on case reports only [30, 31]).

### Supportive care

Transfusion of red blood cells is necessary when hemolysis causes severe anemia, but the optimal transfusion threshold is not determined. Platelet transfusions are considered to aggravate platelet aggregation and disturbance of microcirculation, and current guidelines give a 1 A recommendation to avoid platelet transfusions unless there is life-threatening hemorrhage [7]. Intensive care treatment is often necessary in patients with severe organ damage. Neurologic deterioration or brain ischemia may require sedation and mechanical ventilation, antiepileptic therapy and an experienced intensive care team (stroke unit). Acute coronary syndrome requires hemodynamic monitoring, myocardial or circulatory support, or coronary interventions. Renal failure requires careful fluid and electrolyte management and often renal replacement. During all these sophisticated medical treatments, PEX and specific TMA treatment need to be continued, which may cause logistic challenges. Prophylaxis of venous thromboembolism is necessary and recommended even with lower platelet counts [7]. Low molecular weight heparins are usually used, monitoring of dose is necessary in patients with renal dysfunction.

### New/approaching therapeutic options for TTP

#### Anti-VWF agents: ALX-0081 (caplacizumab; Cablivi®)

Caplacizumab is a bivalent nanobody, recombinantly composed from the functional fragments of the variable domains of the heavy-chain-only immunoglobulins from camelidae (llama). It is designed to bind specifically and with a high affinity to the A1 domains of VWF, the physiological ligands of platelet receptors GP Ib/IX. Thus, caplacizumab competes with platelet binding and therefore inhibits platelet aggregation and activation, but does not affect collagen binding or ADAMTS13 susceptibility of VWF. Caplacizumab can be administered subcutaneously and causes suppression of VWF function for up to 48 h after a single dose [32]. Caplacizumab has been studied two randomized controlled trials (TITAN [33, 34], EudraCT 2010-019375-30 and HERCULES [35], EudraCT 2015-001098-42) in patients with acute autoimmune TTP, and has been proven to reduce the time to platelet normalization, organ damage, exacerbations, health care resource utilization and mortality. The drug is available via an early access program; it will soon be approved by the authorities under the brand name Cablivi®.

#### Recombinant ADAMTS13

The current development of recombinant ADAMTS13 (rADAMTS13) as a therapeutic agent to replace for the missing enzyme will probably be a great advancement in the treatment of congenital TTP [36]. The possibility to avoid plasma therapy with all the disadvantages (time-consuming, thawing, large fluid volume, potential risk of pathogen transmission, side effects, immunogenicity, allergic reaction) and to establish prophylactic or therapeutic home-treatment (like in hemophiliacs) will be a clear improvement of the current situation. Clinical trials with rADAMTS13 in congenital TTP are in progress and approval can hopefully be expected within the next few years [37]. Due to the long half-life and the low necessary plasma concentration of ADAMTS13 prophylactic therapy in Upshaw–Shulman syndrome will probably be sufficient with 20–40 U/kg every 2–4 weeks, depending on the results of the current trials. Dosing for acute bouts needs to be determined, but single or dual shots of the same dose may be sufficient to terminate an episode. There is, however, the considerable risk of inducing anti-ADAMTS13 alloantibodies during treatment (like in hemophiliacs with FVIII inhibitors), which will cause major problems, as neither plasma infusion nor PEX or rADAMTS13 will be sufficient to treat acute bouts of TTP.

#### N-acetylcysteine

N-acetylcysteine (NAC) is an antioxidative substance that has already been clinically used as a mucolytic agent for many years [38]. In models using human plasma, purified VWF and ADAMTS13 knock-out mice it could be demonstrated that NAC leads to a reduction of UL-VWF MM by disrupting the disulfide bonds in the A1 domain of VWF [39]. This also leads to a reduced binding of platelets to VWF released from endothelial cells [39]. Considering the pathophysiology of TTP, cleavage of the UL-VWF MM by a substance with low side effects would be advantageous. One recent case report that showed promising results in a patient with refractory TTP treated with NAC has been published [40]. Thus, a clinical trial with NAC in addition to PEX for the treatment of TTP has been started and is currently recruiting (N-acetylcysteine in TTP. U.S. National Institutes of Health http://clinicaltrials.gov/show/NCT01808521).

## Concluding remarks

Considerable progress has been made in diagnosis and therapy of TTP. New assays to measure ADAMTS13 activity and anti-ADAMTS13 antibodies can establish the diagnosis (distinguish between congenital and autoimmune TTP and the different other types of TMA) within a few hours and are useful to guide treatment. Consequent PEX therapy clearly has improved survival. Congenital TTP responds well to the replacement of ADAMTS13 by plasma infusion. Autoimmune types of TTP often respond to PEX, immunosuppression and rituximab.

The near future will probably bring a variety of other therapeutic possibilities: blocking the VWF-platelet interaction with anti-VWF A1 agents, replacement of ADAMTS13 by a recombinant ADAMTS13 concentrate, cleavage of VWF with NAC, or better immunosuppressive strategies. However, the low incidence of TTP clearly reduces the possibility to perform randomized controlled trials with a sufficient number of patients. Even in ongoing worldwide trials recruitment is poor, thus, making the development of a new drug expensive and time-consuming. This encourages the treating physicians to use off-label treatments when the therapeutic principle fits in the pathophysiological understanding of the disease. The severity of the disease and the high risk of developing permanent damage clearly justifies such an approach in some situations.

## References

[1] Moschcowitz E (1924). Hyaline thrombosis of the terminal arterioles and capillaries: A hitherto undescribed disease. Proc NY Pathol Soc.

[2] Moake JL, Rudy CK, Troll JH (1982). Unusually large plasma factor VIII: Von Willebrand factor multimers in chronic relapsing thrombotic thrombocytopenic purpura. N Engl J Med.

[3] Furlan M, Robles R, Galbusera M (1998). Von Willebrand factor-cleaving protease in thrombotic thrombocytopenic purpura and the hemolytic-uremic syndrome. N Engl J Med.

[4] Tsai HM, Lian EC (1998). Antibodies to von Willebrand factor-cleaving protease in acute thrombotic thrombocytopenic purpura. N Engl J Med.

[5] Knöbl PN (2013). Treatment of thrombotic microangiopathy with a focus on new treatment options. Haemostaseologie.

[6] Scully M, Hunt BJ, Benjamin S (2012). British Committee for Standards in Haematology. Guidelines on the diagnosis and management of thrombotic thrombocytopenic purpura and other thrombotic microangiopathies. Br J Haematol.

[7] Fontana S, Kremer Hovinga JA, Lämmle B (2006). Treatment of thrombotic thrombocytopenic purpura. Vox Sang.

[8] Chapman K, Seldon M, Richards R (2012). Thrombotic microangiopathies, thrombotic thrombocytopenic purpura, and ADAMTS-13. Semin Thromb Hemost.

[9] Lämmle B, Kremer Hovinga JA, Alberio L (2005). Thrombotic thrombocytopenic purpura. J Thromb Haemost.

[10] Tripodi A, Peyvandi F, Chantarangkul V (2008). Second international collaborative study evaluating performance characteristics of methods measuring the von Willebrand factor cleaving protease (ADAMTS-13). J Thromb Haemost.

[11] Kokame K, Kokubo Y, Miyata T (2011). Polymorphisms and mutations of ADAMTS13 in the Japanese population and estimation of the number of patients with Upshaw-Schulman syndrome. J Thromb Haemost.

[12] Miyata T, Kokame K, Matsumoto M, Fujimura Y (2013). ADAMTS13 activity and genetic mutations in Japan. Haemostaseologie.

[13] Fujimura Y, Matsumoto M, Isonishi A, Yagi H, Kokame K, Soejima K, Murata M, Miyata T (2011). Natural history of Upshaw-Schulman syndrome based on ADAMTS13 gene analysis in Japan. J Thromb Haemost.

[14] Mansouri Taleghani M, von Krogh AS, Fujimura Y, George JN, Hrachovinová I, Knöbl PN (2013). Hereditary thrombotic thrombocytopenic purpura and the hereditary TTP registry. Hamostaseologie.

[15] Scheiflinger F, Knöbl P, Trattner B (2003). Nonneutralizing IgM and IgG antibodies to von Willebrand factor-cleaving protease (ADAMTS-13) in a patient with thrombotic thrombocytopenic purpura. Blood.

[16] Tsai HM (2012). Autoimmune thrombotic microangiopathy: Advances in pathogenesis, diagnosis, and management. Semin Thromb Hemost.

[17] Schaller M, Studt JD, Voorberg J, Kremer Hovinga JA (2013). Acquired thrombotic thrombocytopenic purpura. Development of an autoimmune response. Haemostaseologie.

[18] Ferrari S, Knöbl P, Kolovratova V (2012). Inverse correlation of free and immune complex-sequestered anti-ADAMTS13 antibodies in a patient with acquired thrombotic thrombocytopenic purpura. J Thromb Haemost.

[19] Ferrari S, Palavra K, Gruber B (2013). Persistence of circulating ADAMTS13-specific immune complexes in patients with acquired thrombotic thrombocytopenic purpura. Haematologica.

[20] Mariotte E, Azoulay E, Galicier L (2016). Epidemiology and pathophysiology of adulthood-onset thrombotic microangiopathy with severe ADAMTS13 deficiency (thrombotic thrombocytopenic purpura): A cross-sectional analysis of the French national registry for thrombotic microangiopathy. Lancet Haematol.

[21] Knöbl P (2014). Inherited and acquired thrombotic thrombocytopenic purpura (TTP) in adults. Semin Thromb Hemost.

[22] Rock GA, Shumak KH, Buskard NA (1991). Comparison of plasma exchange with plasma infusion in the treatment of thrombotic thrombocytopenic purpura. Canadian Apheresis Study Group. N Engl J Med.

[23] Cataland SR, Scully MA, Paskavitz J (2011). Evidence of persistent neurologic injury following thrombotic thrombocytopenic purpura. Am J Hematol.

[24] Falter T, Alber KJ, Scharrer I (2013). Long term outcome and sequelae in patients after acute thrombotic thrombocytopenic purpura episodes. Haemostaseologie.

[25] Deford CC, Reese JA, Schwartz LH (2013). Multiple major morbidities and increased mortality during long-term follow-up after recovery from thrombotic thrombocytopenic purpura. Blood.

[26] Som S, Deford CC, Kaiser ML (2012). Decreasing frequency of plasma exchange complications in patients treated for thrombotic thrombocytopenic purpura-hemolytic uremic syndrome, 1996 to 2011. Transfusion.

[27] Knoebl P, Koder S, Schellongowski P (2008). Monitoring of ADAMTS13 in patients with thrombotic thrombocytopenic purpura: Prediction of response to therapy, risk of relapse, and long-term outcome. Blood.

[28] Froissart A, Buffet M, Veyradier A (2012). Efficacy and safety of first-line rituximab in severe, acquired thrombotic thrombocytopenic purpura with a suboptimal response to plasma exchange. Experience of the French Thrombotic Microangiopathies Reference Center. Crit Care Med.

[29] Scully M, McDonald V, Cavenagh J (2011). A phase 2 study of the safety and efficacy of rituximab with plasma exchange in acute acquired thrombotic thrombocytopenic purpura. Blood.

[30] Bobbio-Pallavicini E, Porta C, Centurioni R (1994). Vincristine sulfate for the treatment of thrombotic thrombocytopenic purpura refractory to plasma-exchange. The Italian Cooperative Group for TTP. Eur J Haematol.

[31] Beloncle F, Buffet M, Coindre JP (2012). Splenectomy and/or cyclophosphamide as salvage therapies in thrombotic thrombocytopenic purpura: The French TMA Reference Center experience. Transfusion.

[32] Bartunek J, Barbato E, Heyndrickx G, Vanderheyden M, Wijns W, Holz JB (2013). Novel antiplatelet agents: aLX-0081, a nanobody directed towards von Willebrand factor. J Cardiovasc Transl Res.

[33] Peyvandi F, Scully M, Kremer Hovinga JA (2016). Caplacizumab for acquired thrombotic thrombocytopenic purpura. N Engl J Med.

[34] Peyvandi F, Scully M, Kremer Hovinga JA, Knöbl P, Cataland S, De Beuf K, Callewaert F, De Winter H, Zeldin RK (2017). Caplacizumab reduces the frequency of major thromboembolic events, exacerbations and death in patients with acquired thrombotic thrombocytopenic purpura. J Thromb Haemost.

[35] Scully M, Cataland SR, Peyvandi F, Coppo P, Knöbl P, Kremer Hovinga JA (2017). Results of the randomized, double-blind, placebo-controlled, phase 3 Hercules study of Caplacizumab in patients with acquired thrombotic thrombocytopenic purpura. Blood.

[36] Schiviz A, Wuersch K, Piskernik C (2012). A new mouse model mimicking thrombotic thrombocytopenic purpura: correction of symptoms by recombinant human ADAMTS13. Blood.

[37] Scully M, Knöbl P, Kentouche K, Rice L, Windyga J, Schneppenheim R (2017). Recombinant ADAMTS-13: First-in-human pharmacokinetics and safety in congenital thrombotic thrombocytopenic purpura. Blood.

[38] Kelly GS (1998). Clinical applications of N‑acetylcysteine. Altern Med Rev.

[39] Chen J, Reheman A, Gushiken FC (2011). N-acetylcysteine reduces the size and activity of von Willebrand factor in human plasma and mice. J Clin Invest.

[40] Li GW, Rambally S, Kamboj J, Reilly S, Moake JL, Udden MM, Mims MP (2013). Treatment of refractory thrombotic thrombocytopenic purpura with N‑acetylcysteine: A case report. Transfusion.

